# Cervical Microbiota Dysbiosis as a Driver of Cervical Incompetence

**DOI:** 10.3390/microorganisms14102388

**Published:** 2026-10-19

**Authors:** Tingting Wang, Jiaqi Shi, Yinyin Wei, Jie Tang, Jiaqi Lu, Hao Zhang

**Affiliations:** 1 The First Affiliated Hospital of Ningbo University, Ningbo 315000, China; 2 School of Marine Sciences, Ningbo University, Ningbo 315832, China; shijiaqi0321@163.com (J.S.); wei156395@163.com (Y.W.); harrytang123@163.com (J.T.); lujiaqi1@nbu.edu.cn (J.L.); zhanghao@nbu.edu.cn (H.Z.)

**Keywords:** cervical incompetence, microbial dysbiosis, driver taxa, diagnosis model

## Abstract

Cervical incompetence (CIC) represents a significant cause of preterm birth globally. Clinical symptoms of CIC are typically delayed until mid-to-late pregnancy, thereby imposing challenges in CIC diagnosis. There is evidence that the microbiota contributes pivotal roles in reproductive health. By comparing the microbial structure and assembly between 30 CIC patients and 30 healthy controls, we found a close correlation between endocervical microbiota dysbiosis and CIC pathogenesis. The CIC group exhibited increased ɑ-diversity and significant alterations in microbial community composition, characterized by decreased relative abundances of Actinobacteria and Firmicutes. Functional characterization revealed a broad reduction in microbial metabolic potential in patients with CIC. NetShift network analysis identified 22 pivotal driver taxa, including *Lactobacillus* and *Gardnerella* species that triggered the progression to dysbiosis. Notably, a machine learning diagnostic model achieved 83.3% accuracy in clinical diagnosis based on the abundances of 10 biomarkers. These findings provide critical insights into microbial-mediated regulation of the cervical microenvironment by elucidating how specific microbial members and their metabolic potentials contribute to CIC pathogenesis. The diagnosis model offers a new paradigm for diagnosis using microbial biomarkers and reveals potential therapeutic targets via the intervention of driver taxa, thereby establishing innovative strategies to improve pregnancy outcomes.

## 1. Introduction

The human microbiota forms a fundamental component of diverse microenvironments throughout the body, playing indispensable roles in host immune regulation, metabolic balance, and tissue homeostasis [1]. In recent years, mounting evidence has revealed pivotal roles of the microbiota in reproductive health [2]. Notably, the stability of the endocervical microbial communities is essential for maintaining the mucosal barrier integrity of the female reproductive tract [3]. Specifically, the reproductive tract microbiota of healthy women is predominantly composed of lactic acid-producing bacteria [3,4,5] that lower the local pH to around 4.0, thereby suppressing the proliferation of potential pathogens [6]. Accordingly, the cervical microbiota functions as both a physical barrier and an immune modulator, constituting a critical line of defense against ascending infections [7]. Disruption of this finely tuned microbial ecosystem—referred to dysbiosis—predisposes the host to a spectrum of urogenital disorders, including cervical incompetence (CIC), bacterial vaginosis (BV) [8], fungal infections, sexually transmitted infections (STIs) [9], pelvic inflammatory disease (PID) [7], and human immunodeficiency virus (HIV) infection [10]. Recent progress has underscored the importance of microbial homeostasis in sustaining cervical integrity during pregnancy [11,12]. Conversely, dysbiosis in endocervical microbial communities can provoke inflammation, compromise epithelial barrier function, and ultimately contribute to adverse outcomes such as preterm birth or spontaneous miscarriage. Understanding the ecological landscape of the cervical microbiome therefore holds significant implications for maternal health and the prevention of pregnancy complications.

CIC is a pregnancy-associated disorder of the reproductive tract characterized by premature cervical shortening or dilation in the absence of contractions [13]. This condition typically manifests between the second and third trimesters of pregnancy, causing about 10% of preterm deliveries [14]. In cases of late miscarriage, serious complications such as uterine infection or excessive bleeding can occur [15]. Furthermore, CIC poses a high risk of recurrence in subsequent pregnancies [16]. Accumulating evidence supports the hypothesis that bacteria play a pivotal role in the etiology of CIC, especially the dysbiosis in bacterial communities that are significantly correlated with the incidence of preterm birth [17,18,19]. Specific bacterial members, such as *Ureaplasma* and *Mycoplasma* species, could disrupt cervical mucosal integrity, leading to the progression of CIC [20]. However, the mechanistic understanding of these associations of microbial pathogenicity is lacking. It is noteworthy that the symptoms of CIC generally emerge during the mid to late stages of pregnancy, thus challenging early diagnosis [21]. The causes of CIC are complex and varied, with significant differences in etiology and progression among patients [22]. However, current treatments are largely generalized and ignore personalized therapeutic approaches. In addition, the lack of an early diagnosis approach means that many cases are not diagnosed or intervened prior to symptoms appearing [23]. Emerging studies have identified that certain microbial taxa shift significantly before the onset of clinical symptoms, underscoring their potential utility as early biomarkers [19,24,25]. Machine learning models leveraging microbial abundance profiles offer a promising avenue for predicting and diagnosing high-risk individuals, thereby enabling preemptive and tailored interventions. Deciphering the ecological underpinnings of the cervical microbiota and its link to CIC may pave the way for microbiome-informed diagnostic and therapeutic strategies.

Although studies have documented correlations between cervical microbiota composition and CIC incidence [5,11], most remain descriptive and do not elucidate the functional contributions of driver taxa. It is now well understood that microbial taxa contribute unequally to community function; low-abundance but highly connected keystone species exert disproportionate influence on microbial network stability [26,27,28]. Building upon previous research, the present study aimed to systematically identify microbial drivers associated with CIC. To this end, we profiled the cervical microbiota of healthy individuals and CIC patients and employed a novel network-based analytical framework, NetShift [29,30], to pinpoint keystone taxa with the key ecological roles. Furthermore, we used BugBase and PICRUSt2 [31,32] to infer microbial phenotypes and metabolic functions, illustrating functional insights into microbiota-mediated pathogenesis. Beyond microbial profiling, we sought to construct a model for the diagnosis of CIC, with the promise of informing personalized clinical decision-making and timely intervention [33,34]. By delineating the potential relationships between cervical microbiota composition and disease risk, this study lays a foundation for microbiome-guided diagnostics and prevention strategies—contributing to the precision management of maternal health.

## 2. Materials and Methods

### 2.1. Clinical Cohorts and Sample Collection

In 2024, a total of 60 reproductive-aged Chinese women were recruited from the First Affiliated Hospital of Ningbo University, comprising 30 pregnant women clinically diagnosed with CIC and 30 healthy pregnant controls. CIC was clinically diagnosed based on a history of painless cervical dilation followed by second- or third-trimester pregnancy loss or preterm birth, cervical length < 25 mm in the second trimester, or cervical dilation without contractions. Exclusion criteria included vaginal inflammation, malignancies, severe pelvic adhesions, and endocrine or autoimmune disorders. Participants who had received antibiotics, progesterone, or immunosuppressants within the preceding month, undergone cervical procedures or irrigation, or engaged in sexual activity within one month prior to recruitment were also excluded.

All cervical mucus samples were uniformly collected at a gestational age of 18 weeks. A sterile vaginal speculum was used to expose the cervix under direct visualization. A sterile swab was then gently introduced into the endocervical canal and rotated to collect endocervical mucus. Swabs were immediately placed into sterile containers and transported to the laboratory within 2 h after collection, then stored at −80 °C until further analysis. The study protocol and sample collection procedures were approved by the Ethics Committee of the First Affiliated Hospital of Ningbo University (Approval No. 103A-2024).

### 2.2. 16S rRNA Gene Sequencing of the Cervical Microbiota

Total DNA from cervical canal samples was extracted using a BIOG DNA Swab Kit (Changzhou Bio-generating Biotechnology Corp., Changzhou, China) according to the manufacturer’s instructions. DNA concentration was assessed using Qubit 4.0 (Thermo Fisher Scientific, Waltham, MA, USA). DNA purity was evaluated by spectrophotometric measurement of the OD260/280 ratio, and samples with an OD260/280 ratio of 1.8–2.0 were considered acceptable for downstream use. The V3–V4 regions of the 16S rDNA gene were amplified by PCR using primers 341F and 806R (341F: 5′-GTGCCAGCMGCCGCGGTAA-3′; 806R 5′-GGACTACHVGGGTWTCTAAT-3′). To minimize bias during PCR amplification, each sample was amplified in triplicate in a 50 μL reaction system using 50 ng of quality-qualified purified DNA as template. The PCR conditions were as follows: 25 cycles of denaturation at 95 °C for 30 s, annealing at 55 °C for 30 s, and extension at 72 °C for 45 s, followed by a final extension at 72 °C for 10 min. The triplicate PCR products for every sample were combined and purified using a PCR fragment purification kit (TIANGEN Biotech, Beijing, China). Sequencing libraries were constructed from purified PCR amplicons using the TIANSeq Fast DNA Library Prep Kit (Illumina) (TIANGEN Biotech, Beijing, China). The resulting libraries were pooled in equimolar proportions and subjected to paired-end sequencing (2 × 300 bp) on an Illumina MiSeq platform (TIANGEN Biotech, Beijing, China). The raw sequencing data generated in this study have been deposited in the Genome Sequence Archive (GSA) hosted by the National Genomics Data Center (NGDC), China National Center for Bioinformation (CNCB), with the accession numbers PRJCA066950.

### 2.3. Illumina Data Processing

The quality of the sequences was assessed using FastQC (version 0.12.1) [35], and adapters, low-quality bases (average quality score < 20), and fragments shorter than 36 bp were removed using Trimmomatic (version 0.39). Subsequently, usearch (version 11.0.667) was used to remove redundant sequences and singletons. Sequence denoising was performed using the UNOISE3 algorithm (usearch) to generate zero-radius operational taxonomic units (zOTUs) at single-nucleotide resolution. The algorithm corrects sequencing errors based on an error probability model, while simultaneously identifying and removing chimeric sequences. This process ultimately yields zOTU representative sequences and their corresponding abundance matrix at single-nucleotide resolution [36]. Bacterial taxonomic annotation was performed using the RDP Classifier (version 2.14), which employs a k-mer-based naive Bayesian algorithm, against the RDP 16S Training Set v18 reference database. A confidence threshold of 0.8 was applied at each taxonomic rank, and assignments below this threshold were discarded [37]. After excluding non-prokaryote-associated sequences and filtering zOTUs with very low abundance (*n* ≤ 10 across the enrolled 60 samples), we obtained a total of 10,375,572 effective reads and 702 zOTUs in subsequent analysis.

### 2.4. Statistical Analysis

All statistical analyses and visualizations were conducted within the R environment (version 4.3.0). Species rarefaction curves were generated to assess whether the sequencing depth in this study was adequate. Each line represents one sample. The progressively decreasing slopes and tendency toward a plateau indicate that the sequencing effort captured most of the detectable zOTU diversity (Figure S1). Potential outliers were identified using boxplots and verified against the original data, with valid observations retained. The Shapiro–Wilk and Levene tests indicated that the data were approximately normally distributed and satisfied the assumption of homogeneity of variances. Therefore, an unpaired *t*-test was used to compare physiological parameters, including pH, age, BMI, pre-pregnancy weight, cervical canal lengths, neonatal weight, gestational days at delivery, and cervical dilation, between the CIC group and healthy controls. Assessment of alpha diversity relied on rarefied zOTU tables, with Simpson and Shannon indices serving as determinants. Between-group comparisons were performed using the Wilcoxon rank-sum test, and Cliff’s delta was calculated as the effect size. Partial distance-based redundancy analysis (Partial db-RDA/CAP) was performed using Bray−Curtis dissimilarities to assess differences in microbial community composition between CIC patients and healthy controls, with maternal pre-pregnancy weight included as a covariate. Statistical significance was evaluated using permutational multivariate analysis of variance (PERMANOVA, Adonis2) in the R package “vegan”. All data are presented as the mean + standard error of the mean. ns indicates no significant difference, * *p* < 0.05; ** *p* < 0.01; *** *p* < 0.001.

### 2.5. Predicted Functional Potentials of Microbiotas Between Healthy and Cervical Incompetence

To assess the functional capabilities of bacterial communities, the zOTU table underwent normalization by dividing the abundance of each zOTU by its predicted 16S copy number. This procedure facilitates the categorization of functions according to the Kyoto Encyclopedia of Genes and Genomes (KEGG) using the PICRUSt2 (Phylogenetic Investigation of Communities by Reconstruction of Unobserved States) tool within the Galaxy platform (http://huttenhower.org/galaxy, accessed on 20 April 2026) [31]. This methodology was utilized to infer functional potentials between individuals with CIC and healthy controls. To identify metabolic pathways significantly associated with CIC while accounting for potential confounding factors, multivariable differential abundance analyses of functional features were performed using Microbiome Multivariable Associations with Linear Models (MaAsLin2), with disease status as the primary explanatory variable and pre-pregnancy weight included as covariate. The direction and magnitude of the associations were determined from the regression coefficients, with negative coefficients indicating lower predicted pathway abundances in the CIC group relative to healthy controls after adjustment for pre-pregnancy weight.

### 2.6. Identifying the Potential Microbial Drivers of Cervical Incompetence

The zOTUs that drove the bacterial network from health to CIC were identified using a newly established “NetShift” approach [29]. Unlike traditional network analysis, which mainly describes static network structure, NetShift can capture network rewiring between healthy and diseased states and identify low-abundance but topologically important driver taxa. This approach enables the quantification of alterations in interspecies associations and the escalation in node importance through NESH (Neighbor Shift) scores and ΔB (delta betweenness), reflecting the frequency of node occurrence in paths connecting other nodes. The two indices precisely assess node associations, facilitating the quantitative identification of “driver” taxa within microbial networks, distinguishing between “diseased” and “healthy” states [29]. Specifically, a taxon in a diseased state displaying modified associations (manifested by a high NESH value) while concurrently exhibiting increased network importance (evidenced by a positive ΔB) holds pivotal significance in microbial interactions and is designated as a “driver”. To retain robust correlations and minimize noise from weak associations, a correlation coefficient threshold of |R| > 0.7 was applied to define significant microbial associations. Additionally, the NESH score captures directional alterations in nodes, indicative of exclusive enrichment in disease-related interacting partners over healthy counterparts, thus facilitating the identification of events such as pathogen colonization [29]. Within this framework, “driver” taxa could serve as candidate pathogens underlying CIC.

### 2.7. Construction of Diagnosis Model for Diagnosing Cervical Incompetence

A diagnosis model was constructed to distinguish CIC individuals from healthy controls using the “RandomForest” package in R [33]. To minimize potential demographic bias, zOTUs significantly associated with maternal pre-pregnancy weight in the healthy control group were excluded prior to model construction. The samples were divided into training and independent test sets, and a nested cross-validation strategy (five outer folds and ten inner folds) was implemented within the training set to optimize model performance. Specifically, within each outer training fold, a random forest model comprising 500 decision trees was initially constructed, and feature importance was evaluated using mean decrease in accuracy (MDA) [38]. Candidate microbial features were then selected based on 100 permutation tests (*p* < 0.05). Subsequently, random forest recursive feature elimination (rfcv), combined with ten-fold inner cross-validation, was employed to determine the optimal number of features corresponding to the lowest cross-validation error. Model performance was evaluated using the held-out validation subset in each outer fold. After aggregating the results across the five outer folds, the final number of features was determined based on the minimum mean cross-validation error. A final random forest model comprising 500 decision trees was then trained using the entire training set and evaluated on the independent test set to assess its classification performance. The top 10 microbial features contributing to the classification task were selected based on their permutation importance scores.

## 3. Results

### 3.1. Clinical Characteristics

**Figure 1:**
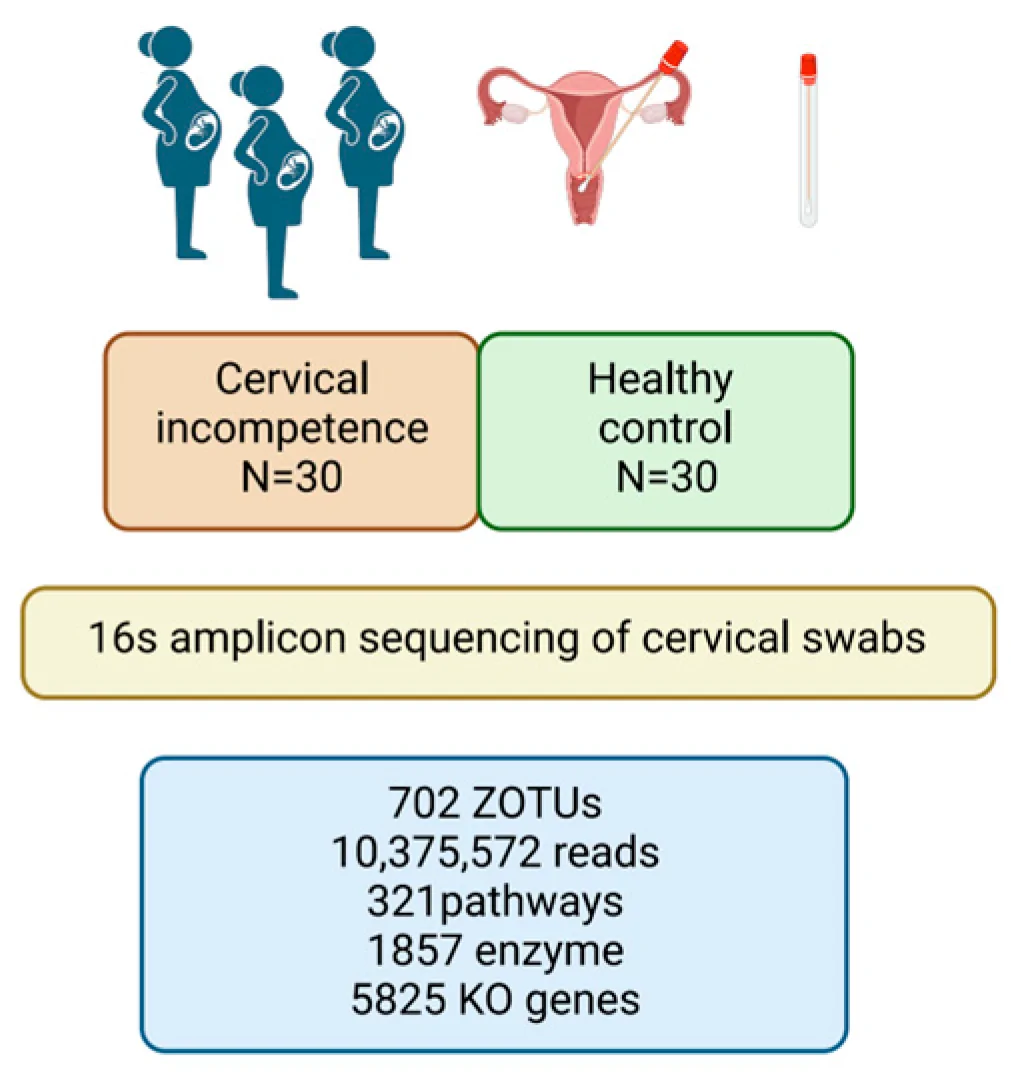
Study design and the profiles of the cervical microbiomes.

**Figure 2:**
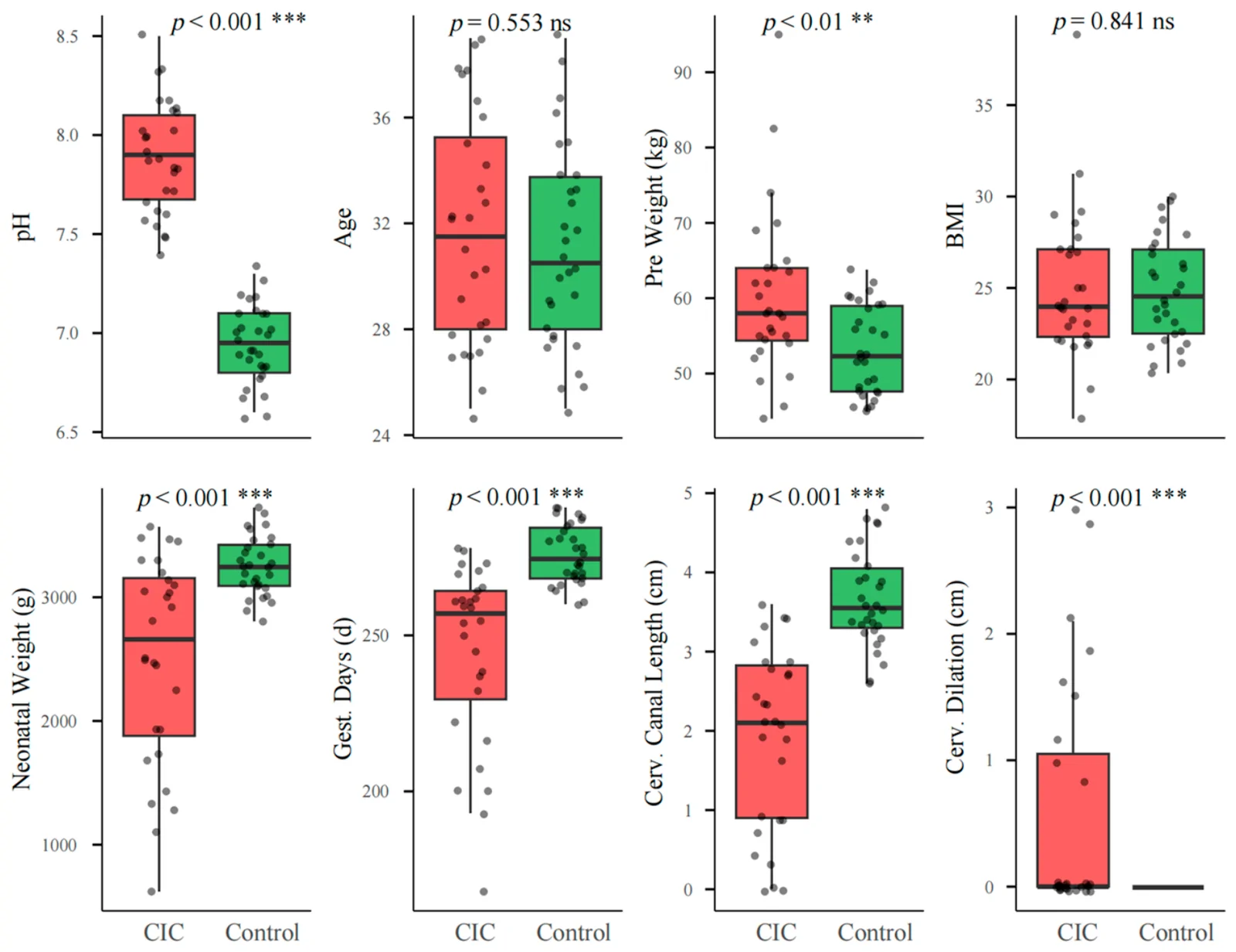
Physiological comparison between CIC patients and healthy pregnant controls using unpaired *t*-test.

A total of 60 pregnant women in their second or third trimesters were enrolled in this study, including 30 patients clinically diagnosed with CIC and 30 healthy pregnant women as controls (Figure 1). Healthy controls ranged in age from 25 to 39 years, with a mean of 32.5 years, which was comparable to the CIC group (Table S2), thereby ruling out human age effect on the cervical microbiome. Their pre-pregnancy weights ranged from 48.2 to 65.0 kg, and gestational weight gain ranged from 9.3 to 14.9 kg, resulting in delivery weights of 59.2 to 79.3 kg. BMI values at sampling mostly fell between 21.0 and 26.6. Endocervical pH values were mildly alkaline, ranging from 6.6 to 7.3. Cervical canal lengths in the control group were typically between 3.6 and 4.8 cm, and the cervical dilation was completely closed in all cases. Gestational days at delivery ranged from 259 to 288 days (equivalent to 37 + 0 to 41 + 1 weeks), and neonatal weight ranged from 2860 to 3570 g. The majority of the healthy controls delivered vaginally. In contrast, the CIC group demonstrated a tendency toward higher pre-pregnancy weights and greater gestational weight gain (Table S1). Cervical canal lengths were substantially shortened (as low as 0.3 cm), and cervical dilation was significantly increased (up to 3.0 cm in some cases). In addition, patients with CIC exhibited overall shorter gestational durations, a higher prevalence of preterm delivery, and generally lower neonatal weights (Figure 2). Statistical analysis confirmed significant between-group differences in pH (*p* < 0.001), pre-pregnancy weight (*p* < 0.01), cervical canal length (*p* < 0.001), cervical dilation (*p* < 0.001), gestational days at delivery (*p* < 0.001), and neonatal weight (*p* < 0.001). No significant differences were observed in maternal age (*p* = 0.481) or BMI (*p* = 0.792) (Figure 2).

### 3.2. Alterations of Microbial Community Structure in Cervical Incompetence

**Figure 3:**
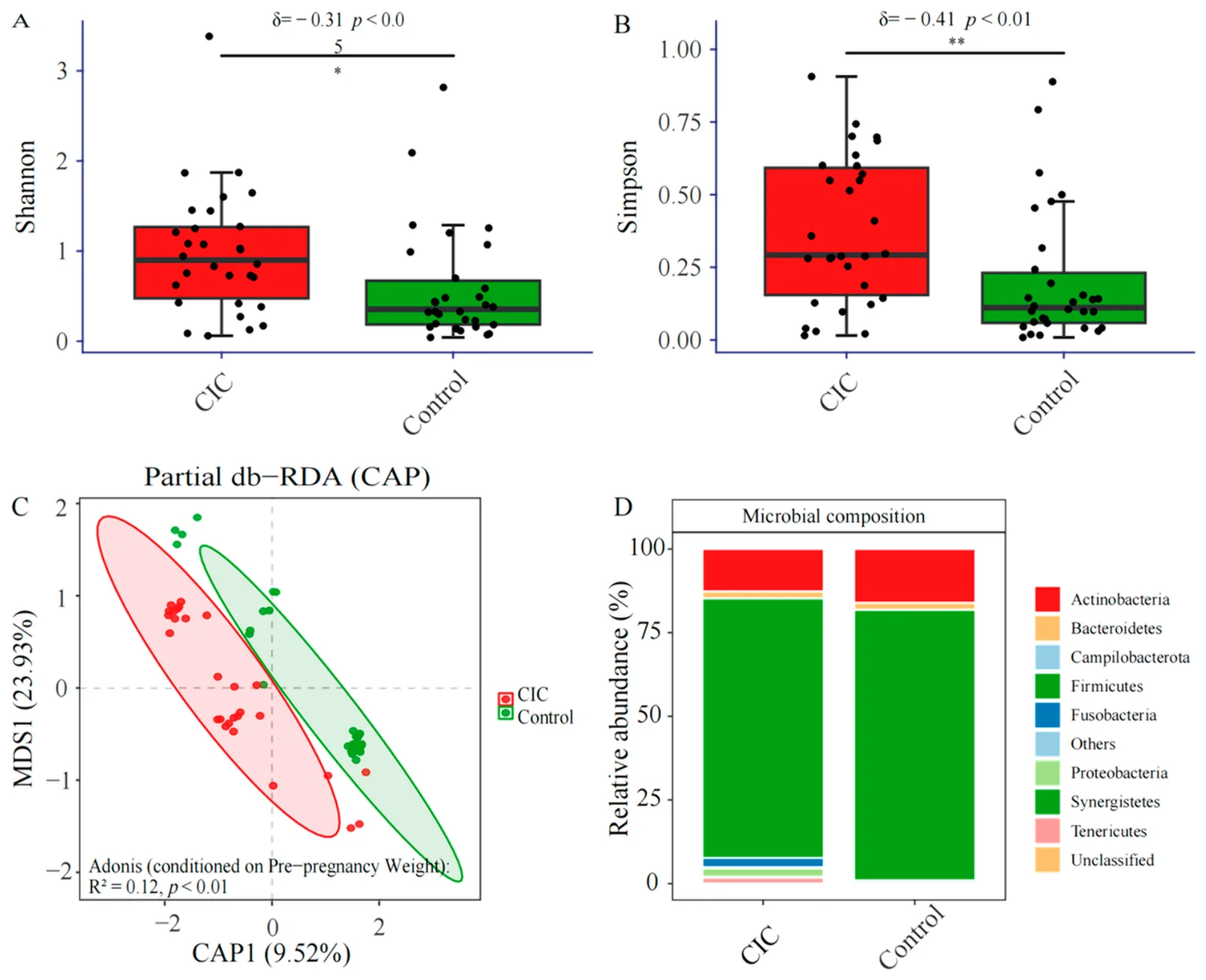
Comparative analysis of cervical microbiome diversity and composition between patients with CIC and healthy controls. (**A**) Shannon diversity index: Box plots depicting the distribution of Shannon index values, showing higher microbial diversity in the CIC group compared to controls, with statistical significance (*p* < 0.05) using Wilcoxon rank-sum test. (**B**) Simpson diversity index: Box plots showing the Simpson index values, indicating significantly greater microbial diversity in the CIC group (*p* < 0.01). (**C**) Partial db-RDA/CAP based on Bray−Curtis dissimilarities, illustrating differences in cervical microbial community composition between CIC patients and healthy controls after adjusting for maternal pre-pregnancy weight. Statistical significance was assessed using PERMANOVA. (**D**) Microbial composition relative abundance: Bar graph displaying the relative abundance of bacterial phyla in the CIC group compared to health control group.

Cervical swab samples were collected from the enrolled participants to characterize the cervical microbiome. After quality control, a total of 702 zOTUs were obtained. Functional prediction based on KEGG annotation revealed 321 metabolic pathways, 1857 enzymes, and 5825 KEGG orthologous (KO) genes (Figure 1). The median Shannon index in the CIC group was significantly higher (Cliff’s δ = −0.31, *p* < 0.05) than that in the control group (Figure 3A). Similarly, the Simpson index also reflected higher diversity in the CIC group, with statistical significance noted (Cliff’s δ = −0.41, *p* < 0.01) (Figure 3B). Partial db-RDA/CAP revealed significant differences in cervical bacterial community composition between CIC patients and healthy controls after adjusting for pre-pregnancy weight (PERMANOVA, R^2^ = 0.12, *p* < 0.01; Figure 3C). In the CIC group, higher proportions of Bacteroidetes (unpaired *t* test, *p* < 0.05) were noted, whereas Actinobacteria and Firmicutes were more abundant in the control group (Figure 3D). In contrast, Proteobacteria and Fusobacteria were rare between the two groups, with a slight increase in unclassified bacteria in the CIC group (Figure 3D).

Overall, there are substantial disparities in the diversity and composition of the cervical microbiome between patients with CIC and healthy individuals, which may be linked to the pathophysiological processes underlying cervical insufficiency.

### 3.3. Functional and Phenotypic Analysis of CIC Microbiome

**Figure 4:**
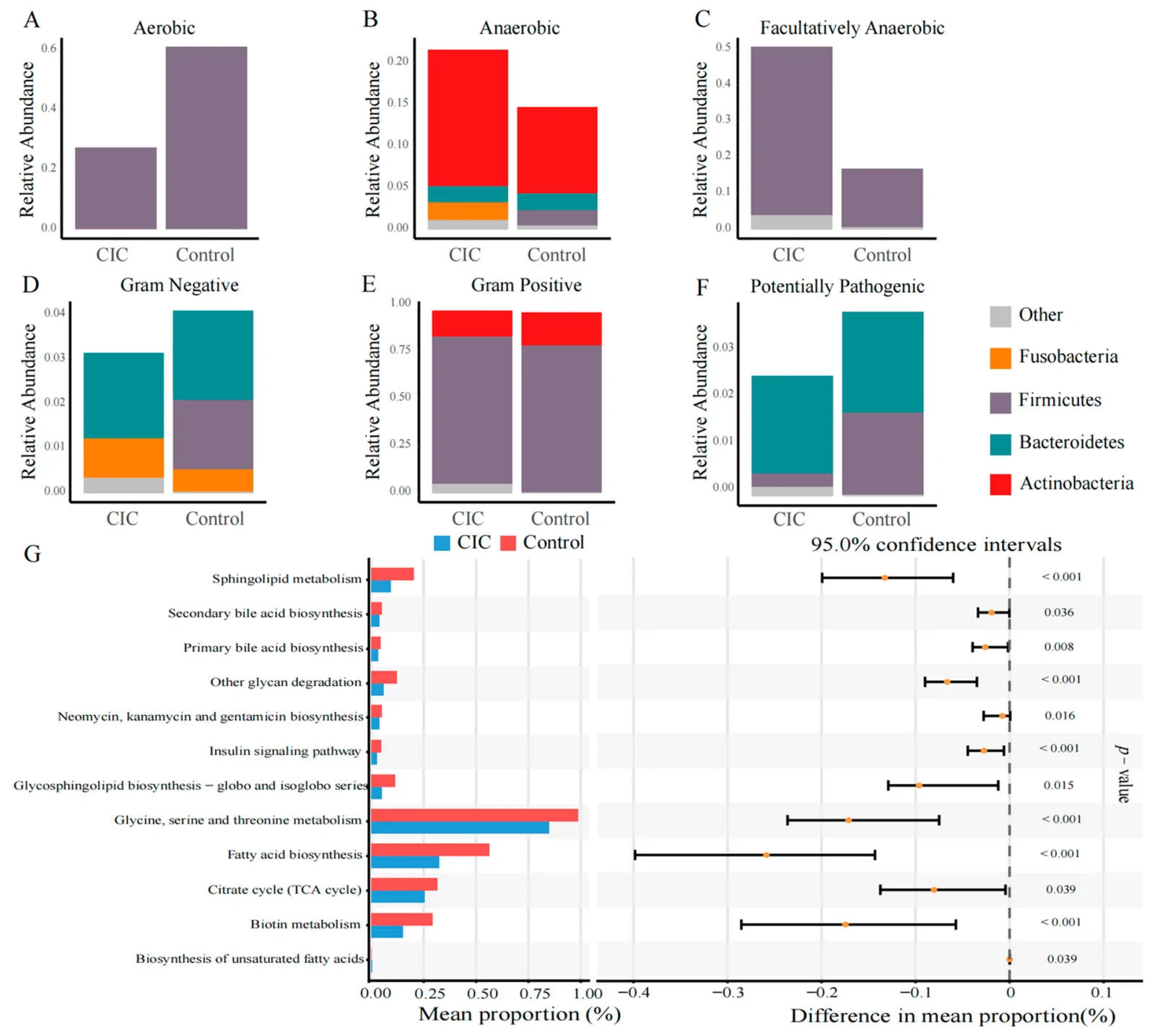
Comparison of phenotypic and functional structures between CIC and healthy cohorts. (**A**–**F**) BugBase-predicted relative abundances of aerobic, anaerobic, facultatively anaerobic, Gram-negative, Gram-positive, and potentially pathogenic bacteria. (**G**) PICRUSt2-predicted metabolic pathways and their mean abundance differences between groups, with 95% confidence intervals. Differential pathway associations were assessed using MaAsLin2, adjusting for maternal pre-pregnancy weight.

BugBase analysis revealed variations in microbial phenotypes between the CIC group and the healthy control group (Figure 4A–E). Specifically, aerobic microbes exhibited a higher proportion in the control group (0.61) than in the CIC group (0.27, Figure 4A), indicating that a reduction in aerobic microbial populations could be associated with CIC. In contrast, the CIC group posed a higher relative abundance of anaerobic microbes (0.21), particularly Actinobacteria (Figure 4B). Facultatively anaerobic microbes were more prevalent in the CIC group, indicating an altered microbial ecological balance associated with CIC (0.51 vs. 0.17 in control group, Figure 4C). Additionally, the control group (0.04) had a higher relative abundance of Gram-negative bacteria, such as Fusobacteria, which was less abundant in the CIC group (0.03, Figure 4D). Conversely, the CIC group (0.97 vs. 0.96 in control group) exhibited a higher relative abundance of Gram-positive bacteria (Figure 4E).

The PICRUSt2 analysis inferred notable differences in metabolic pathways between the CIC and control groups (Figure 4G). Specifically, the pathways for glycine, serine, and threonine metabolism, glycolysis, the citric acid cycle, sphingolipid metabolism, secondary bile acid biosynthesis, and fatty acid metabolism were consistently depressed in the CIC patients. These results indicate that the metabolic state of the CIC group could shift towards a more energy-conserving and stress-adaptive metabolic profile.

### 3.4. Identification of CIC Driver Taxa

**Figure 5:**
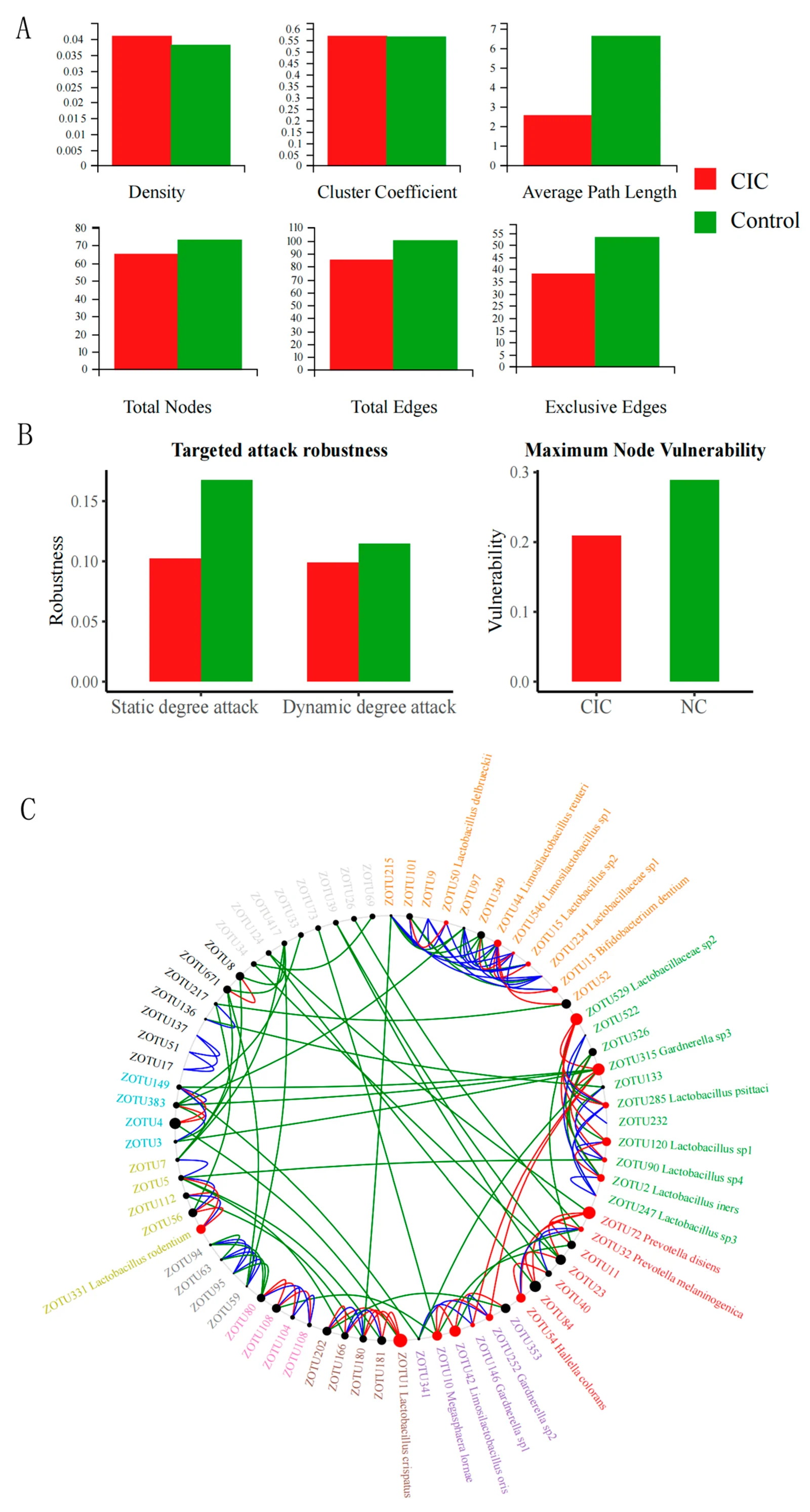
NetShift analysis identifies driver taxa shifting microbial network from healthy to CIC. (**A**) Network topological properties showing changes between Control and CIC samples. (**B**) Network robustness and vulnerability analysis. Network robustness was assessed under static and dynamic degree-based targeted attacks. Static degree attack removes nodes according to their initial degree, whereas dynamic degree attack recalculates node degree after each removal step. Maximum node vulnerability represents the greatest impact of single-node removal on network efficiency or connectivity. (**C**) The common sub-network is extracted, and nodes are arranged by community membership. The size of each node is proportional to its NESH score, which indicates the extent of their association shift. Red nodes highlight taxa with increased intermediation in the CIC state, marking them as significant “drivers.” Black nodes represent taxa present in both states but interacting with the common sub-network differently in CIC and Control.

As shown in Figure 5A, we compared the topological features of the bacterial networks between the CIC group and the control group. Compared to the control group, the CIC group exhibited a notably shorter average path length (2.5 vs. 6.5), suggesting more direct and efficient information transmission within the CIC network. Additionally, the CIC group showed lower values of total nodes (67 vs. 75), total edges (83 vs. 98), and exclusive edges (38 vs. 52) than the control group, implying a simplified network structure under the CIC condition. Both groups displayed similar network density (approximately 0.035) and cluster coefficient (approximately 0.63), with slightly higher values in the CIC group.

The CIC network robustness values were lower than those of the control network under both targeted attack modes (static degree attack: 0.10 vs. 0.17; dynamic degree attack: 0.10 vs. 0.12, Figure 5B), indicating that the CIC network had a weaker ability to maintain its overall connected structure after the removal of highly connected nodes. Therefore, CIC network architecture was more sensitive to the loss of key nodes and exhibited reduced overall resistance to perturbation. Further analysis of the most vulnerable node showed that the maximum node vulnerability was higher in the control network than that in the CIC network (0.29 vs. 0.21, Figure 5B), indicating that the control network contained a key node with a greater impact on network efficiency or connectivity.

Figure 5C illustrates the common sub-network, in which nodes were arranged by community membership, with similar colors indicating the same community. Driver taxa, highlighted as larger red nodes, exhibited significant changes in their associations, thereby acting as “drivers” in the transition from a healthy to a diseased status. A total of 22 driver taxa belonging to seven genera were identified: *Lactobacillus*, *Bifidobacterium*, *Gardnerella*, *Hallella*, *Limosilactobacillus*, *Megasphaera*, and *Prevotella* (Figure S2, Table S3).

### 3.5. Establishment of Diagnosis Model for Diagnosing CIC

**Figure 6:**
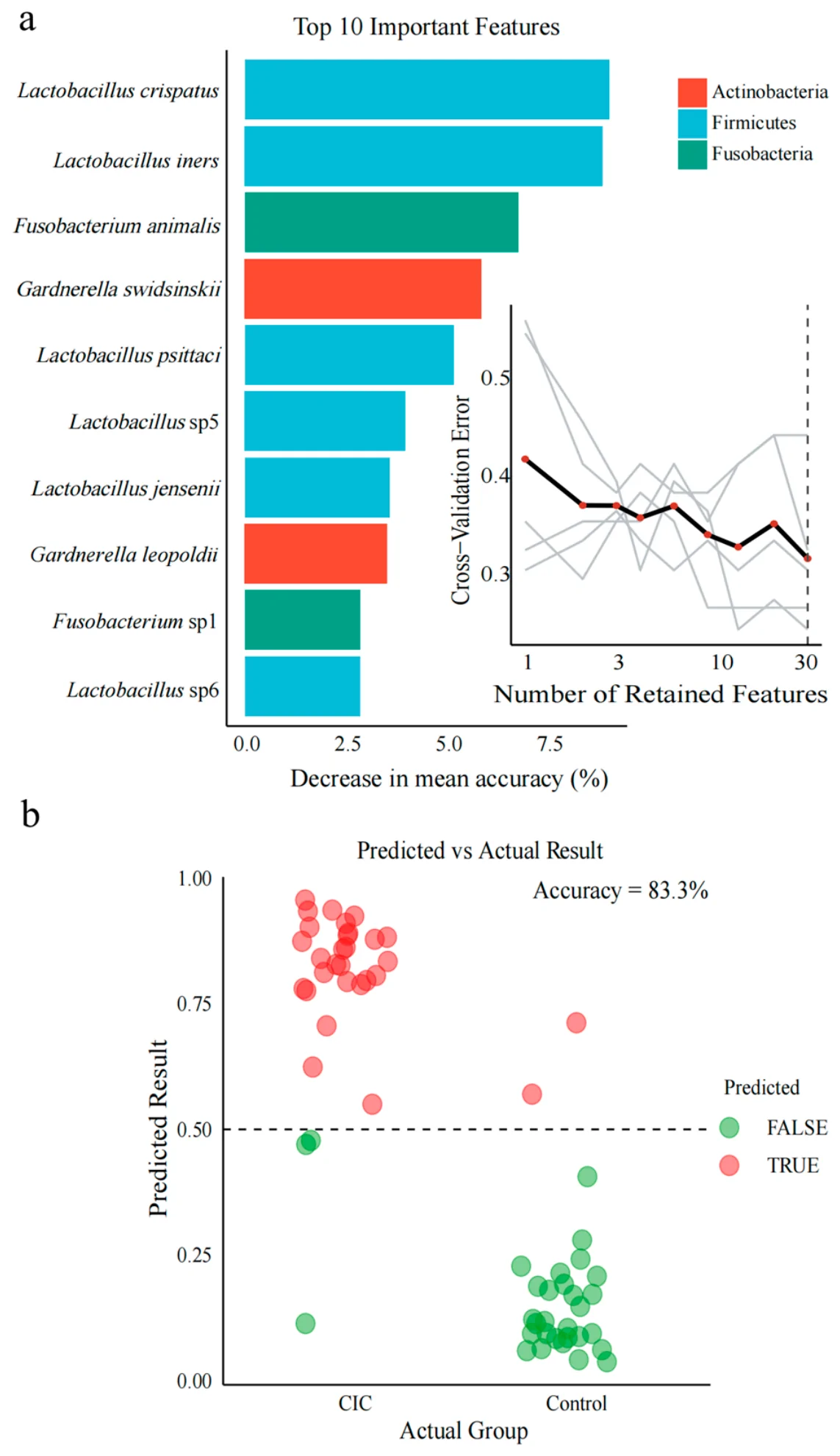
Predictive analysis of CIC based on cervical microbiome using Random Forest model. (**a**) Importance ranking of the top 10 microbial features based on mean decrease in accuracy (MDA). The inset shows cross-validation error curves across different numbers of retained features, with gray lines representing individual outer folds and the black line indicating the mean error. The dashed vertical line indicates the optimal number of features. (**b**) Scatter plot showing predicted CIC probabilities across the CIC and control groups. Red and green dots represent predicted CIC probabilities above and below the classification threshold of 0.5, respectively. The reported overall classification accuracy was 83.3%.

To diagnose CIC status, we developed a random forest classifier to evaluate the diagnostic potential of microbial markers for CIC by comparing 30 CIC patients with 30 healthy controls. A nested cross-validation strategy was employed to optimize feature selection and model performance. The cross-validation error curve showed that the lowest mean error (0.31) was achieved when approximately 30 zOTUs were retained (Figure 6a). The top 10 microbial features were ranked according to their mean decrease in accuracy (MDA), with zOTUs putatively assigned to *Lactobacillus crispatus*, *Lactobacillus iners*, and *Fusobacterium animalis* exhibiting the highest importance scores (Figure 6a and Figure S2, Table S4). The random forest model achieved a reported overall classification accuracy of 83.3% (Figure 6b), demonstrating the potential of cervical microbial features to distinguish patients with established CIC from healthy controls. These findings suggest that microbial signatures may have potential clinical utility for CIC classification at 18 weeks of gestation.

## 4. Discussion

CIC remains a major challenge in pregnancy management, particularly in the context of preterm birth prevention [39,40]. While CIC is traditionally viewed as an anatomical defect, emerging evidence has indicated that cervical microbial homeostasis disruption is implicated in its etiology. However, the underlying mechanisms driving this process remain poorly understood [41,42,43]. To address this gap, we integrated 16S rRNA sequencing, NetShift network analysis, and machine learning to pinpoint taxa with key ecological roles and diagnostic power. We observed pronounced microbial imbalance in CIC patients (Figure 3 and Figure 4). Network analysis showed that *Lactobacillus* and *Gardnerella* were the keystone nodes driving the transition to a pathological state (Figure 5C). Furthermore, a random forest model based on microbial markers achieved an overall 83.3% accuracy, suggesting its potential clinical utility for CIC risk stratification at 18 weeks of gestation. (Figure 6b).

Unlike the gut ecosystem, where a high diversity is a hallmark of health, the healthy cervical microbiome relies on low diversity and *Lactobacillus* dominance to maintain homeostasis [44,45]. *Lactobacillus* species maintain a low pH microenvironment by producing lactic acid, thereby inhibiting the colonization of low-pH-tolerant pathogens and preserving mucosal defense functions [46]. In this study, driven by the depletion of dominant Firmicutes (primarily protective *Lactobacillus*), and the concurrent enrichment of Actinobacteria and Bacteroidetes, the Shannon and Simpson indices in the CIC group significantly increased compared with the healthy controls (Figure 3A). Specifically, the cervical microbiota under CIC conditions is not dominated by a single beneficial population (e.g., *Lactobacillus* species) but rather exhibits a highly heterogeneous community structure characterized by increased diversity of detrimental *Gardnerella* and *Fusobacterium* species (Figure 3D and Figure 6a). This pattern mirrors the “Anna Karenina principle”: healthy endocervical communities share a convergent, *Lactobacillus*-dominated stability, whereas CIC-associated communities display diversified dysbiosis [47]. Importantly, here the significantly higher pH observed in the CIC group was in line with previous reports showing that depletion of *Lactobacillus* population is often associated with reduced acidification capacity, elevated local pH, impaired mucosal barrier function, and enhanced inflammatory responses (Figure 2) [42,48,49]. Collectively, dysbiosis in the cervical microbiota could increase the risk of cervical shortening and CIC-related phenotypes by weakening the acidic barrier and exacerbating inflammatory responses.

To identify keystone taxa triggering the transition of the state from health to CIC, we applied the NetShift network analysis [30]. Topological analysis revealed reductions in the numbers of nodes and edges in the CIC network compared with the healthy network (Figure 5A). The decreased network robustness renders the cervical microbiota that is more susceptible to the invasion by exogenous pathogens (Figure 5B) [50]. The significantly shortened average path length in the CIC network (Figure 5A) suggests an enhanced efficiency of information transfer and communication among network members [50,51]. In pathological conditions, this increased network efficiency could facilitate the rapid spread of pathogens or pro-inflammatory signals that accelerate disease progression [52,53]. Beyond alterations in the overall topological structure, we also identified *Lactobacillus* and *Gardnerella* species as keystone nodes. Specifically, the protective *Lactobacillus* exhibited reduced dominance and decreased network centrality, whereas the dysbiosis-associated *Gardnerella* was markedly enhanced in the CIC network (Figure 5C). Therefore, the occurrence of CIC could be triggered by changes in a few keystone nodes, rather than a synchronous shift in the entire microbial community [43,54]. Consistently, alterations in key nodes could reduce the network stability [55,56,57], thereby compromising the ability of the cervical microecosystem to resist ascending infections and amplifying local inflammation, and consequently promoting the development of CIC.

Consistent with previous observations that compositional shifts in the cervical microbiota in CIC are accompanied by functional alterations [58], our study found that the cervical microecosystem in CIC also underwent metabolic reprogramming. PICRUSt2 analysis revealed a broad reduction in microbial metabolic potential in patients with CIC. Specifically, pathways involved in central carbon and energy metabolism—including glycolysis, the tricarboxylic acid cycle, and glycine, serine, and threonine metabolism—were significantly depressed (Figure 4G). This metabolic suppression is likely driven by the marked depletion of *Lactobacillus* species, which is a predominant genus in the healthy cervical microbiota. *Lactobacilli* strains are known to ferment carbohydrates (primarily glycogen) into lactic acid via glycolysis, thereby maintaining a low endocervical pH and creating a hostile environment for pathogens [58,59,60]. By this logic, the reduction in *Lactobacillus* abundance could directly decrease the enzymatic flux through glycolytic pathways, leading to diminished downstream tricarboxylic acid cycle activity and reduced availability of amino acid-derived metabolites (e.g., glycine, serine, and threonine), as supported by lowered pH in CIC individuals (Figure 2). Consequently, a *Lactobacilli*-dominated, metabolically acidic defense state shifts to a diverse but low-energy, stress-adaptive CIC state. Such an energy-constrained microbial milieu may influence epithelial homeostasis and local immune tone by altering metabolite availability, which increases cervical tissue vulnerability [61,62,63]. Concurrently, the predicted lipid-related functions were also suppressed, particularly pathways associated with sphingolipid metabolism and the biosynthesis of unsaturated fatty acids (Figure 4G). These altered potentials in lipid metabolism could, in turn, modulate microbe–host interactions relevant to membrane-associated processes and barrier integrity, leading to impaired cervical epithelial barrier function [62,63], and subsequent increased susceptibility to external perturbations and ascending infections [64,65]. Collectively, CIC-associated dysbiosis is accompanied by dysfunctions in acid production, energy, and lipid metabolic potentials, which causes the occurrence of CIC by affecting cell membrane stability, immune responses, and tissue repair processes in the cervix.

Currently, the clinical diagnosis of CIC primarily relies on transvaginal ultrasound measurements of cervical length during mid-to-late pregnancy. However, this index essentially reflects structural phenotypic changes that have already occurred, which results in a delay in detecting earlier pathological processes [66,67]. Existing studies have shown that microbial dysbiosis often precedes detectable histological changes [68,69,70], suggesting the potential value of microbial markers for early disease detection. Accordingly, the combination of 10 key microbial markers achieved a classification accuracy of 83.3% using a random forest model (Figure 6), demonstrating the feasibility and potential clinical utility of microbial features for CIC risk stratification at 18 weeks of gestation. Based on these findings, incorporating microbial marker screening into prenatal assessment protocols could provide warning signals, thereby assisting clinicians in identifying high-risk individuals and optimizing intervention decisions before significant cervical shortening occurs.

Despite the encouraging findings, several limitations of this study should be acknowledged. First, V3–V4 amplicon sequencing has limited resolution at the bacterial species level, and mock communities and negative controls were not included in the experimental design. Second, while functional predictions based on 16S rRNA gene sequencing provide valuable insights, these inferences rely on reference databases and model assumptions, making it difficult to achieve strain-level resolution and confirm the expression of specific metabolic genes [71,72]. Additionally, the cross-sectional design only reveals the close association between cervical microbiome dysbiosis and CIC, without clarifying whether the dysbiosis is a cause of the disease or a consequence of cervical shortening [73]. Future studies should employ full-length 16S sequencing or shotgun metagenomics to achieve higher taxonomic resolution at the species and strain levels, and focus on large-scale, multicenter prospective longitudinal cohorts that allow for the systematic tracking of microbiome dynamics from early pregnancy onward. Furthermore, integrating metagenomic and metabolomic analyses would enable a more direct characterization of microbial functional activity and help validate the causal roles and underlying mechanisms of identified candidate driver taxa.

## 5. Conclusions

In summary, this study presents a new theoretical framework based on systems microbiology, revealing the driving taxa, biomarkers, and metabolic adaptation mechanisms underlying CIC. We propose that CIC is driven by the depletion of keystone taxa—specifically the replacement of *Lactobacillus* by *Gardnerella* species—which triggers a cascade of network metabolic suppression. Changes in network homeostasis compromise cervical barrier and fuels inflammation. Particularly, we identify 10 biomarkers that achieve a high accuracy in diagnosing CIC from healthy individuals. These findings not only deepen our understanding of the relationship between the reproductive tract microbiome and pregnancy outcomes but also lay the theoretical foundation for the future development of microbiome-based precise diagnostic tools and targeted intervention strategies.

## Data Availability

The raw sequencing data generated in this study have been deposited in the Genome Sequence Archive (GSA) hosted by the National Genomics Data Center (NGDC), China National Center for Bioinformation (CNCB), with the accession numbers PRJCA066950. These data are publicly accessible via the GSA database (https://ngdc.cncb.ac.cn/gsa/ (accessed on 18 June 2026)). All other relevant data supporting the findings of this study are included in the article or available from the corresponding author upon reasonable request.

## Supplementary Materials

The following supporting information can be downloaded at: https://www.mdpi.com/article/10.3390/microorganisms14102388/s1, Figure S1. Rarefaction curves of the cervical microbiota samples. Figure S2. Phylogenetic validation of taxonomic assignments for representative zOTUs. Table S1: Clinical and demographic characteristics of healthy control pregnant women in the study population. Table S2: Clinical and demographic characteristics of pregnant women in the study population. Table S3. Sequence-based taxonomic identification of representative zOTUs used in the NetShift analysis. Table S4. Sequence-based taxonomic identification of representative zOTUs used in the Random Forest analysis.
