# Supplementary material for: Cervical Microbiota Dysbiosis as a Driver of Cervical Incompetence
Source: Microorganisms. 2026 Oct 19;14(10):2388. doi: 10.3390/microorganisms14102388 (PMC13707414; doi:10.3390/microorganisms14102388)
Supplement: Supplementary file 1 [file microorganisms-14-02388-s001.zip › Supplementary Figure S1.pdf]

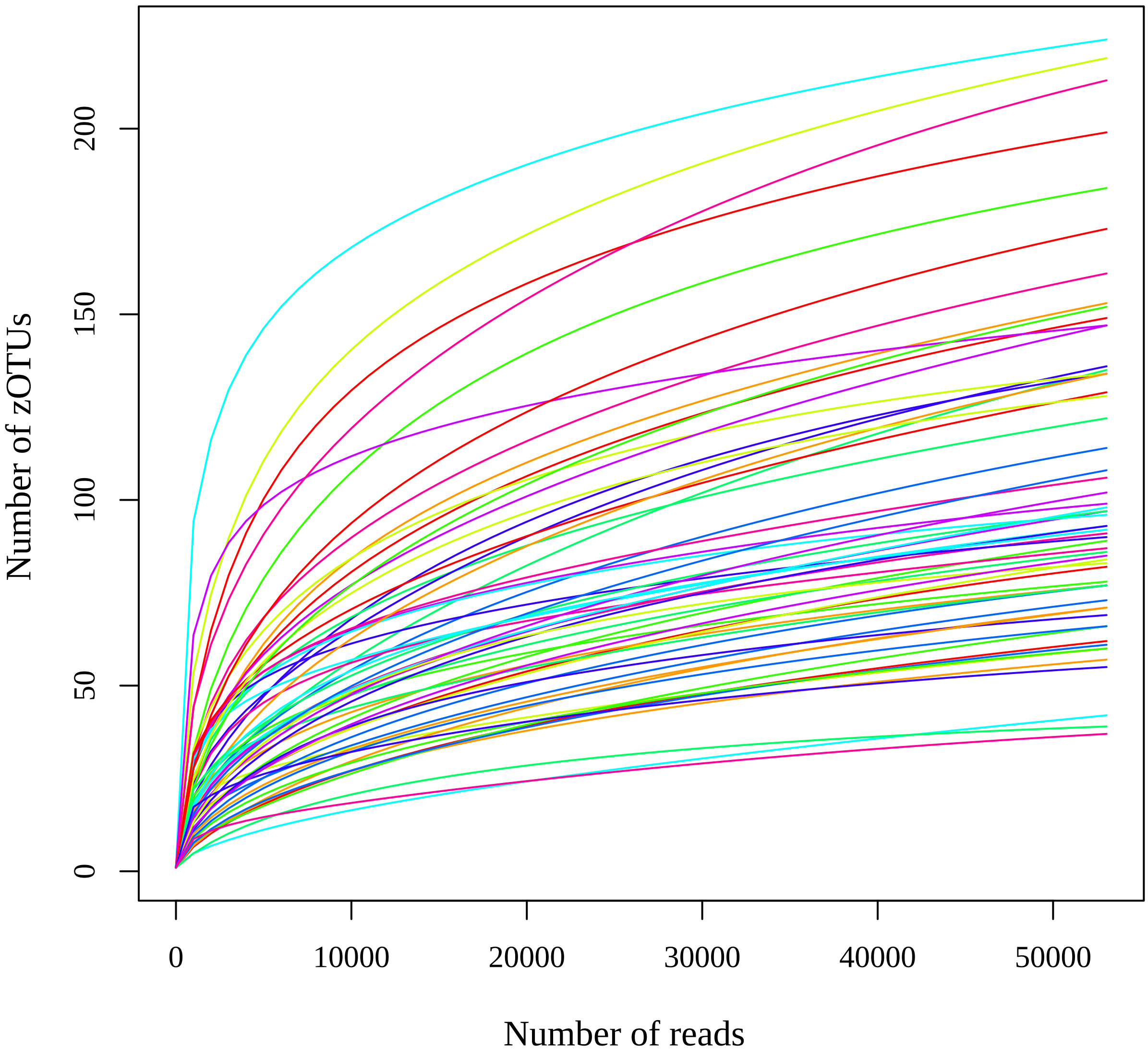

Number of zOTUs
0
50
100
150
200
Number of reads
0
10000
20000
30000
40000
50000
