# Supplementary material for: Cervical Microbiota Dysbiosis as a Driver of Cervical Incompetence
Source: Microorganisms. 2026 Oct 19;14(10):2388. doi: 10.3390/microorganisms14102388 (PMC13707414; doi:10.3390/microorganisms14102388)
Supplement: Supplementary file 1 [file microorganisms-14-02388-s001.zip › Supplementary Figure S2.pdf]

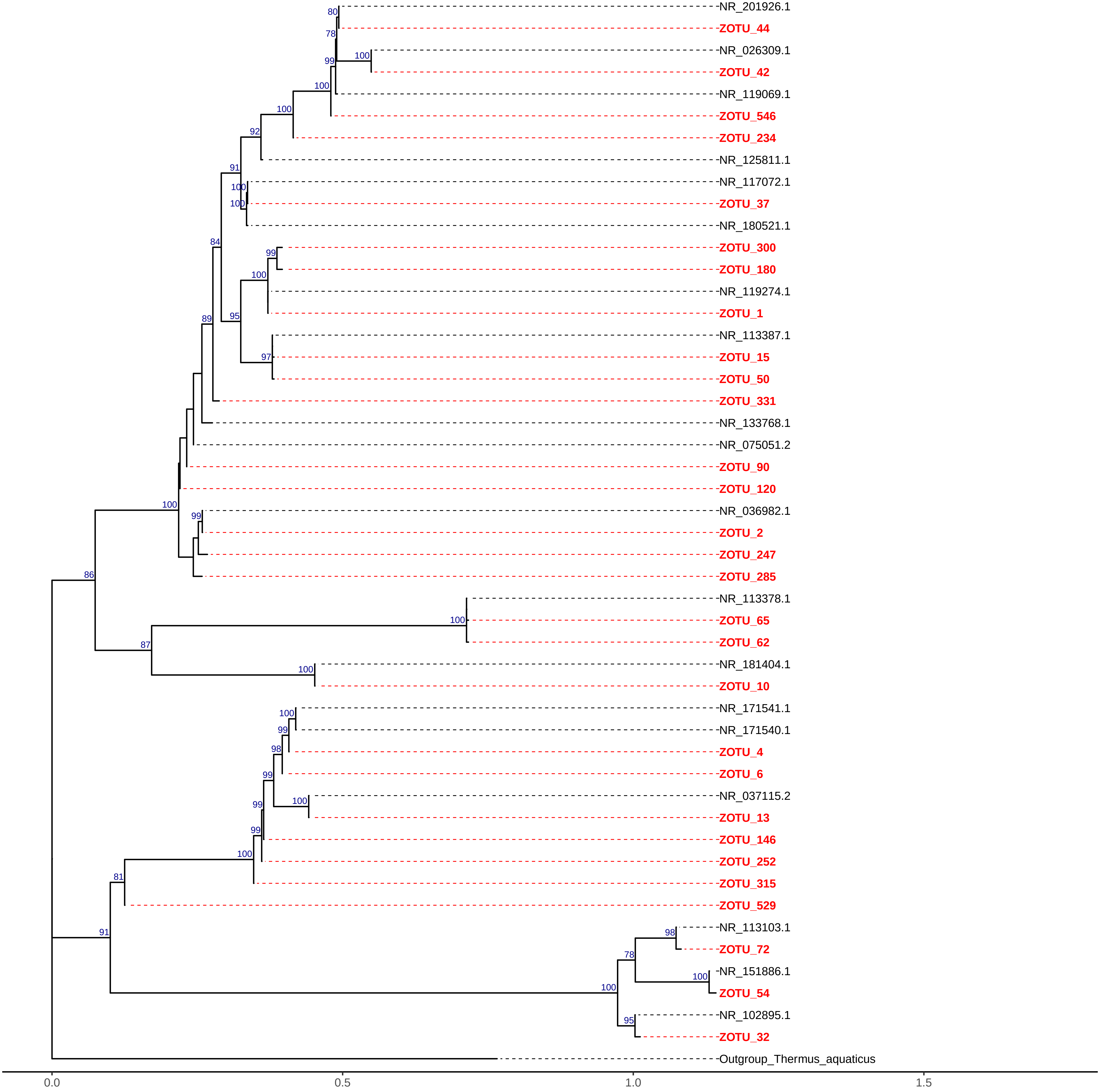
NR_201926.1
ZOTU_44
NR_026309.1
ZOTU_42
NR_119069.1
ZOTU_546
ZOTU_234
NR_125811.1
NR_117072.1
ZOTU_37
NR_180521.1
ZOTU_300
ZOTU_180
NR_119274.1
ZOTU_1
NR_113387.1
ZOTU_15
ZOTU_50
ZOTU_331
NR_133768.1
NR_075051.2
ZOTU_90
ZOTU_120
NR_036982.1
ZOTU_2
ZOTU_247
ZOTU_285
NR_113378.1
ZOTU_65
ZOTU_62
NR_181404.1
ZOTU_10
NR_171541.1
NR_171540.1
ZOTU_4
ZOTU_6
NR_037115.2
ZOTU_13
ZOTU_146
ZOTU_252
ZOTU_315
ZOTU_529
NR_113103.1
ZOTU_72
NR_151886.1
ZOTU_54
NR_102895.1
ZOTU_32
Outgroup_Thermus_aquaticus
0.0
0.5
1.0
1.5
