# Supplementary material for: Cervical Microbiota Dysbiosis as a Driver of Cervical Incompetence
Source: Microorganisms. 2026 Oct 19;14(10):2388. doi: 10.3390/microorganisms14102388 (PMC13707414; doi:10.3390/microorganisms14102388)
Supplement: Supplementary file 1 [file microorganisms-14-02388-s001.zip › Supplementary Table S1.pdf]

Table S1. Clinical and demographic characteristics of healthy control pregnant women in the study population.

ID	pH	Age	Pre-pregnancy Weight (kg)	Weight (kg)	Gain weight (kg)	Bmi	Internal OS Type (T/Y/V/U)	Neonatal Weight (g)	Delivery Type	Gestational Days at Delivery	Cervical Canal Length	Cervical Dilation
H1	6.8	26	55.8	65.7	9.9	22.48	T	3150	Vaginal delivery	288	2.8	2.5
H2	7	28	52.6	64.4	11.8	25.16	Y	3405	Cesarean section	268	4.1	1.3
H3	7.1	33	46.4	61.3	14.9	24.33	U	3260	Cesarean section	265	3.3	2.5
H4	6.7	31	56.8	69.2	12.4	26.07	V	3580	Cesarean section	276	3.5	1.6
H5	7.2	29	47.7	57	9.3	27.45	T	2970	Vaginal delivery	268	3.9	2.5
H6	6.9	27	59.7	70.4	10.7	23.12	Y	3100	Vaginal delivery	264	3.1	1.8
H7	6.6	32	60.1	71.5	11.4	20.89	T	3340	Cesarean section	281	3	2.6
H8	7	30	45	56.1	11.1	21.56	U	3725	Cesarean section	283	3.8	1.9
H9	7.1	34	45.5	58.8	13.3	28.73	V	2890	Vaginal delivery	287	4.6	2.9
H10	6.8	35	48.9	59.2	10.3	25.84	T	3480	Vaginal delivery	269	3.9	1.6
H11	6.9	26	63.8	78.5	14.7	22.60	Y	3250	Vaginal delivery	278	4.4	2.6
H12	7.2	28	58.6	69.3	10.7	26.31	U	2995	Cesarean section	285	3.7	2.7
H13	7.3	33	52.1	66.4	14.3	29.42	T	3360	Vaginal delivery	261	3.3	1.7
H14	6.7	36	45.6	58.2	12.6	23.85	Y	3180	Vaginal delivery	270	3.2	2.7

H15	7	37	47	59.7	12.7	24.75	V	3550	Vaginal delivery	273	4.4	0.3
H16	6.8	25	52.5	64.9	12.4	27.92	U	3075	Cesarean section	278	3.4	1.2
H17	6.9	29	55.9	66.5	10.6	20.34	T	3110	Cesarean section	266	4.6	0.8
H18	7.1	27	51.5	62.3	10.8	25.60	V	3590	Cesarean section	289	3.6	1.1
H19	6.6	38	47.4	59.9	12.5	26.84	Y	3240	Cesarean section	291	3.6	0
H20	7	35	59.2	73.6	14.4	23.28	U	2805	Cesarean section	286	4.8	1.7
H21	6.8	28	59.1	70.4	11.3	21.95	T	3460	Cesarean section	291	3.9	0.8
H22	6.9	30	49.2	63.6	14.4	22.14	Y	3300	Vaginal delivery	267	2.6	1.5
H23	7.2	31	61	70.3	9.3	28.06	U	2955	Vaginal delivery	270	3.5	1.4
H24	7.1	26	62.1	73.3	11.2	24.08	V	3090	Cesarean section	273	2.6	2.5
H25	7	29	51.5	65.8	14.3	29.76	T	3680	Vaginal delivery	280	4.7	1.9
H26	6.7	39	47.6	61.9	14.3	20.72	U	3010	Cesarean section	260	4.2	1.8
H27	7.3	30	55.2	66.5	11.3	27.19	Y	3125	Cesarean section	270	3.4	2.1
H28	6.9	32	60.3	70.8	10.5	23.61	T	3430	Cesarean section	289	3.4	0.2
H29	6.8	34	48.2	59	10.8	21.78	V	3275	Vaginal delivery	272	3.2	0.2
H30	7	33	45.4	56	10.6	30.00	Y	3190	Vaginal delivery	281	3.3	1.6
