# Supplementary material for: Cervical Microbiota Dysbiosis as a Driver of Cervical Incompetence
Source: Microorganisms. 2026 Oct 19;14(10):2388. doi: 10.3390/microorganisms14102388 (PMC13707414; doi:10.3390/microorganisms14102388)
Supplement: Supplementary file 1 [file microorganisms-14-02388-s001.zip › Supplementary Table S2.pdf]

Table S2. Clinical and demographic characteristics of pregnant women in the study population.

ID	pH	Age	Pre-pregnancy Weight (kg)	Weight (kg)	Gain weight (kg)	Bmi	Internal OS Type (T/Y/V/U)	Neonatal Weight (g)	Delivery Type	Gestational Days at Delivery	Cervical Canal Length	Cervical Dilation
C1	7.6	32	49	51	2	21.79	Y	1680	Cesarean section	222	1.9	0
C2	7.8	27	58.3	63.3	5	23.25	Y	3000	Vaginal delivery	273	0.7	1.9
C3	7.5	38	54.5	57	2.5	21.99	T	3300	Cesarean section	261	2.7	0
C4	8	33	62	64	2	25.00	T	2250	Cesarean section	255	2.9	0
C5	7.9	36	49.6	54.6	5	21.87	Y	3140	Cesarean section	265	2.3	0
C6	7.7	39	54	58	4	22.38	T	3480	Vaginal delivery	270	2.7	0
C7	8.2	27	58	60	2	24.03	U	3300	Vaginal delivery	278	0	1.6
C8	7.4	38	60.3	70.3	10	27.12	U	1930	Vaginal delivery	237	0.9	1.2
C9	8	28	53	70	17	27.76	U	2810	Vaginal delivery	254	0.3	2.9
C10	8.1	35	56	59	3	24.24	Y	1330	Cesarean section	200	0.9	2.1
C11	7.6	32	55	58	3	23.83	V	2450	Vaginal delivery	259	2.4	0
C12	7.5	31	55.5	58	2.5	22.10	Y	620	Vaginal delivery	168	2.1	0
C13	8.3	34	74	77.5	3.5	29.17	T	1730	Cesarean section	216	3.4	0
C14	7.9	30	62	62	0	23.05	T	2470	Vaginal delivery	250	2.3	0
C15	8.4	28	78	80	2	27.04	U	/	/	146	1.1	2.1

C16	7.8	25	45.6	48.6	3	19.47	Y		3100	Cesarean section	261	2.1	0.8
C17	7.7	39	64	64	0	25.00	T		2920	Vaginal delivery	271	3.4	0
C18	8.1	28	55	55	0	22.89	T		3470	Vaginal delivery	277	3.3	0
C19	8	38	58	62	4	23.92	T		3570	Cesarean section	262	3.6	0
C20	7.5	32	57.5	61	3.5	27.11	Y		2490	Vaginal delivery	245	2.1	0
C21	8.2	30	95	97	2	38.86	T		3200	Cesarean section	264	2.8	0
C22	7.6	29	64	78	14	31.25	Y		1280	Vaginal delivery	200	0.4	0
C23	7.9	37	82.5	82.5	0	28.55	T		3450	Vaginal delivery	273	3.1	0
C24	8.1	26	69	69	0	26.95	Y		2510	Vaginal delivery	238	0.9	0
C25	8	33	63.5	65	1.5	23.88	Y		3035	Cesarean section	259	1.6	1.5
C26	7.7	28	65	73	8	26.81	T		1930	Cesarean section	232	1.9	0
C27	8.3	27	44	44	0	17.85	T		3050	Vaginal delivery	261	2.9	0
C28	8.5	27	52	54	2	22.19	/		1100	Vaginal delivery	193	0	1
C29	7.8	28	70	78	8	29.00	/		1430	Vaginal delivery	207	0	3
C30	7.6	39	58.1	59	0.9	24.56	/	/		/	161	0	3
