# Supplementary material for: Cervical Microbiota Dysbiosis as a Driver of Cervical Incompetence
Source: Microorganisms. 2026 Oct 19;14(10):2388. doi: 10.3390/microorganisms14102388 (PMC13707414; doi:10.3390/microorganisms14102388)
Supplement: Supplementary file 1 [file microorganisms-14-02388-s001.zip › Supplementary Table S3.pdf]

Table S3. Sequence-based taxonomic identification of representative zOTUs used in the NetShift analysis.

OTU	Identity	Accession	NCBI.name	Name
ZOTU_1	429/429	NR_119274.1	*Lactobacillus crispatus*	*Lactobacillus crispatus*
ZOTU_2	429/429	NR_036982.1	*Lactobacillus iners*	*Lactobacillus iners*
ZOTU_10	429/429	NR_181404.1	*Megasphaera lornae*	*Megasphaera lornae*
ZOTU_13	413/413	NR_037115.2	*Bifidobacterium dentium*	*Bifidobacterium dentium*
ZOTU_15	428/429	NR_113387.1	*Lactobacillus delbrueckii*	*Lactobacillus* sp2
ZOTU_32	424/424	NR_102895.1	*Prevotella melaninogenica*	*Prevotella melaninogenica*
ZOTU_42	429/429	NR_026309.1	*Limosilactobacillus oris*	*Limosilactobacillus oris*
ZOTU_44	429/429	NR_201926.1	*Limosilactobacillus reuteri*	*Limosilactobacillus reuteri*
ZOTU_50	428/429	NR_113387.1	*Lactobacillus delbrueckii*	*Lactobacillus delbrueckii*
ZOTU_54	423/424	NR_151886.1	*Hallella colorans*	*Hallella colorans*
ZOTU_72	424/424	NR_113103.1	*Prevotella disiens*	*Prevotella disiens*
ZOTU_90	425/429	NR_075051.2	*Lactobacillus gasseri*	*Lactobacillus* sp4
ZOTU_120	421/429	NR_075051.2	*Lactobacillus gasseri*	*Lactobacillus* sp1
ZOTU_146	406/412	NR_171541.1	*Gardnerella leopoldii*	*Gardnerella* sp1
ZOTU_234	418/431	NR_180521.1	*Lactobacillus mulieris*	Lactobacillaceae sp1
ZOTU_247	422/429	NR_036982.1	*Lactobacillus iners*	*Lactobacillus* sp3
ZOTU_252	405/412	NR_171541.1	*Gardnerella leopoldii*	*Gardnerella* sp2
ZOTU_285	420/429	NR_125811.1	*Lactobacillus psittaci*	*Lactobacillus psittaci*
ZOTU_315	401/412	NR_171541.1	*Gardnerella leopoldii*	*Gardnerella* sp3
ZOTU_331	420/429	NR_133768.1	*Lactobacillus rodentium*	*Lactobacillus rodentium*
ZOTU_529	281/283	NR_036982.1	*Lactobacillus iners*	Lactobacillaceae sp2
ZOTU_546	425/429	NR_119069.1	*Limosilactobacillus reuteri*	*Limosilactobacillus* sp1
