# Supplementary material for: Cervical Microbiota Dysbiosis as a Driver of Cervical Incompetence
Source: Microorganisms. 2026 Oct 19;14(10):2388. doi: 10.3390/microorganisms14102388 (PMC13707414; doi:10.3390/microorganisms14102388)
Supplement: Supplementary file 1 [file microorganisms-14-02388-s001.zip › Supplementary Table S4.pdf]

Table S4. Sequence-based taxonomic identification of representative zOTUs used in the Random Forest analysis.

OTU	Identity	Accession	NCBI.name	Name
ZOTU_1	429/429	NR_119274.1	*Lactobacillus crispatus*	*Lactobacillus crispatus*
ZOTU_2	429/429	NR_036982.1	*Lactobacillus iners*	*Lactobacillus iners*
ZOTU_65	406/407	NR_113378.1	*Fusobacterium animalis*	*Fusobacterium animalis*
ZOTU_4	411/411	NR_171540.1	*Gardnerella swidsinski*	*Gardnerella swidsinski*
ZOTU_6	412/412	NR_171541.1	*Gardnerella leopoldii*	*Gardnerella leopoldii*
ZOTU_285	420/429	NR_125811.1	*Lactobacillus psittaci*	*Lactobacillus psittaci*
ZOTU_37	429/429	NR_117072.1	*Lactobacillus jensenii*	*Lactobacillus jensenii*
ZOTU_62	405/407	NR_113378.1	*Fusobacterium animalis*	*Fusobacterium sp1*
ZOTU_300	422/429	NR_119274.1	*Lactobacillus crispatus*	*Lactobacillus* sp6
ZOTU_180	421/429	NR_119274.1	*Lactobacillus crispatus*	*Lactobacillus* sp5
